# Improvement of Bacilysin Production in *Bacillus subtilis* by CRISPR/Cas9-Mediated Editing of the 5’-Untranslated Region of the *bac* Operon

**DOI:** 10.4014/jmb.2209.09035

**Published:** 2022-12-13

**Authors:** Hadeel Waleed Abdulmalek, Ayten Yazgan-Karataş

**Affiliations:** 1 Dr. Orhan Ocalgiray Molecular Biology, Biotechnology and Genetics Research Center (ITU-MOBGAM), Istanbul Technical University, Maslak, Istanbul 34469, Turkey; 2 Faculty of Science and Letters, Department of Molecular Biology and Genetics, Istanbul Technical University, Maslak, Istanbul 34469, Turkey; 3 Biotechnology Department, Collage of Science, University of Baghdad, Baghdad, Iraq

**Keywords:** *Bacillus subtilis*, bacilysin, *bacABCDEF*, ribosome binding site, CRISPR/Cas9

## Abstract

Bacilysin is a dipeptide antibiotic composed of L-alanine and L-anticapsin produced by certain strains of *Bacillus subtilis*. Bacilysin is gaining increasing attention in industrial agriculture and pharmaceutical industries due to its potent antagonistic effects on various bacterial, fungal, and algal pathogens. However, its use in industrial applications is hindered by its low production in the native producer. The biosynthesis of bacilysin is mainly based on the *bacABCDEF* operon. Examination of the sequence surrounding the upstream of the *bac* operon did not reveal a clear, strong ribosome binding site (RBS). Therefore, in this study, we aimed to investigate the impact of RBS as a potential route to improve bacilysin production. For this, the 5’ untranslated region (5’UTR) of the *bac* operon was edited using the CRISPR/Cas9 approach by introducing a strong ribosome binding sequence carrying the canonical Shine-Dalgarno sequence (TAAGGAGG) with an 8 nt spacing from the AUG start codon. Strong RBS substitution resulted in a 2.87-fold increase in bacilysin production without affecting growth. Strong RBS substitution also improved the mRNA stability of the *bac* operon. All these data revealed that extensive RBS engineering is a promising key option for enhancing bacilysin production in its native producers.

## Introduction

Bacilysin (also known as bacillin or tetaine) is a non-ribosomally synthesized dipeptide antibiotic comprised of N-terminal L-alanine and C-terminal non-proteinogenic amino acid L-anticapsin through non-thiotemplate mechanism [1-4]. Despite its first discovery in *B. subtilis* [5, 6], bacilysin is also produced by various *Bacillus* species other than *B. subtilis*, including *Bacillus amyloliquefaciens* [7], *Bacillus pumilis*, and *Bacillus velezensis* [8]. Despite its simple structure, bacilysin exhibits a broad range of antimicrobial activity against bacteria and fungi. This antimicrobial activity of bacilysin is conferred by the degradation of its anticapsin moiety upon transport into the cell. Anticapsin inhibits the glucosamine-6-phosphate synthase, and consequently the biosynthesis of the peptidoglycan layer in fungi and bacteria, thereby leading to the lysis of microbial cells [9, 10]. In recent years, plant-associated *Bacillus amyloliquefaciens* subsp. *plantarum* strain FZB42 was shown to suppress the fire blight disease in orchard trees caused by *Erwinia amylovora* as well as rice diseases caused by the bacteria *Xanthomonas oryzae* pv. *oryzae* and *X. oryzae* pv. *oryzicola* through its bacilysin and difficidin secretions [7, 11]. Moreover, *Bacillus amyloliquefaciens* FZB42 exerted intense anticyanobacterial activity against *M. aeruginosa* and other harmful algal species, including A. *flos-aquae*, *Nostoc* sp., and *Anabaena* sp. [12]. Nannan *et al*. [8] reported that bacilysin is a significant player in the antagonistic activity of *B. velezensis* against foodborne pathogens *Escherichia coli* and *Salmonella enterica*. Besides its potent antimicrobial activity, bacilysin is also known to be heat stable (15 min at 100°C) and remains active over a wide range of pH (from 1.4–12.0) [13]. These characteristics make bacilysin a strong candidate for many industrial applications in the pharmaceutical, food, and agricultural industries. However, the main obstacle to its wider industrial application is the low titer of the native producers.

High-level expression of a gene requires more than a high-level transcription. The resulting mRNAs should be effectively translated into proteins. In bacteria, in most cases, translation initiation is the rate-limiting step of translation and a major determinant of the final expression level of a gene [14]. Translation initiation occurs at the ribosome binding site (RBS) located within the 5′ untranslated region (5′UTR) of an mRNA. In bacteria, RBS encloses the Shine-Dalgarno (SD) sequence, the start codon of the target gene, and a short spacer sequence in between. The SD sequence takes a crucial role in translation initiation. Since it enhances the recruitment of the 30S small ribosomal subunit to the start codon through its base pairing with the anti-SD sequence located at the 3′terminus of the 16S rRNA [15]. An SD sequencés effectivity is determined by its base-pairing potential with the anti-SD sequence and its spacing from the translation initiation codon [14, 16, 17]. The canonical SD sequence 5'-UAAGGAGG-3' for most bacterial species, including *B. subtilis*, exhibits the maximum complementation with the anti-SD sequence of 16S rRNA (5’-ACCUCCUUA-3'), the so-called "strong" SD sequence. The SD sequence 5'-UAAGGAGG-3' triggered translation four times more efficiently than the shorter AAGGA sequence [16]. Many consistent studies utilizing reporter genes have demonstrated that increasing the SD-anti-SD interaction improves protein production, emphasizing its significant contribution to the overall translation level [17-19]. The spacer sequence between the SD sequence and start codon is another critical factor affecting translation initiation efficiency. It is positioning too close (< 4 nt) or too far (>14 nt) from the start codon can result in lower levels of initiation. In bacterial genomes, the spacing of the SD generally varies from 5 to 13 nt, with about 8 to 10 nt being ideal for *E. coli* genes [16, 17, 20]. Similarly, the ideal separation in the canonical SD sequence 5'-UAAGGAGG-3' from the AUG start codon was determined to be 7-9 nt for various *B. subtilis* genes [14, 21].

**Fig. 1:**
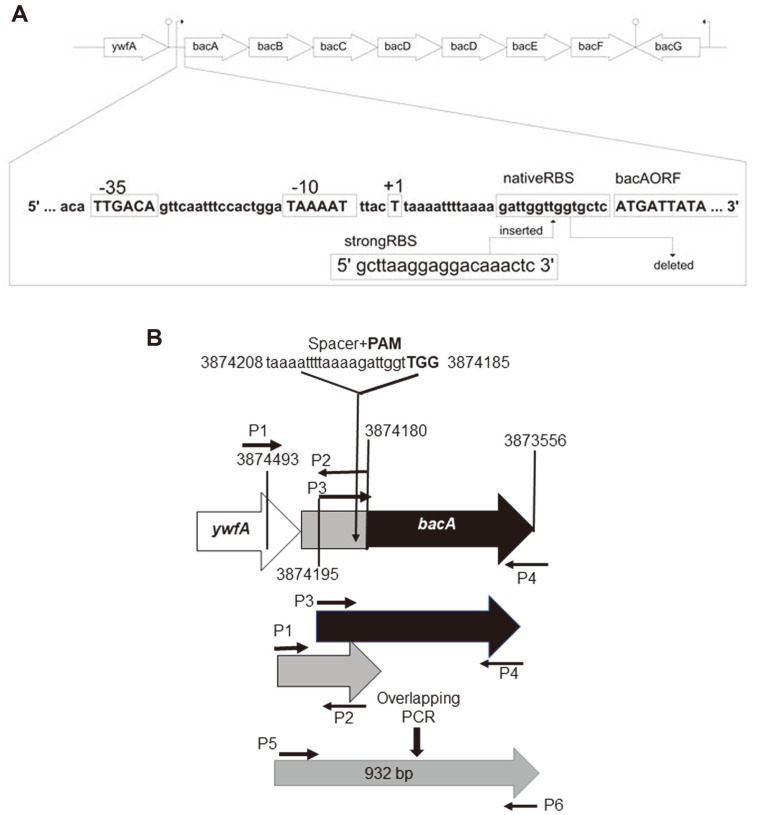
(A) Genomic organization of the *bacABCDEF* operon and the upstream DNA sequences of the *bacA* gene. (B) Schematic representation of the strong RBS substitution region of the *B. subtilis* genome and the construction of the homology template via employing overlap extension PCR.

In *B. subtilis*, the *bacA*BCDEF operon and the neighboring gene *bacG* encode the enzymes responsible for synthesizing anticapsin and bacilysin [22-26]. The transcription of the *bac* operon was shown to be initiated at the T residues 29 bases upstream of the translational start codon [27]. Inspection of the sequence around the *bacA* transcriptional start site revealed a strong consensus signature for the *B. subtilis* sigma factor σ^A^ (TTGACA (-35) and TAAAAT (-10) with a 17-bp spacer) [28] (Fig. 1A). However, there is no obvious strong SD-like sequence surrounding its translation start codon AUG (Fig. 1A), suggesting that improving the RBS region could be a promising key option for enhancing the bacilysin production. Consequently, in this study, we investigated the effect of a strong RBS, which bears the canonical Shine-Dalgarno (SD) sequence (TAAGGAGG) with 8 nt spacing from AUG start codon, on the bacilysin production in *B. subtilis* PY79. For a single gene product, changes in its gene expression level are usually quantified by using a fluorescent reporter protein [14, 18], or by employing a targeted proteomic analysis via a liquid chromatography and mass spectrometry (LC-MS)/MS system [44] to evaluate the effect of an RBS region. However, directly quantifying the translated products could make evaluating the RBS change on a biosynthetic pathway unfeasible since the observed changes in the pathway enzymes usually do not correlate with the observed changes in the final product titer. This is due to confounding factors such as the metabolic burden or the accumulation of toxic pathway intermediates resulting from the changing gene expressions [45]. Consequently, in the scope of this study, we evaluated the effect of potent RBS on the bacilysin biosynthetic pathway with respect to changes in the bacilysin titer, along with its possible effect on the *bac* operon mRNA level. To our knowledge, this is the first report on 5’UTR editing employed on the *bac* operon to boost the bacilysin production in the native producer strain, *B. subtilis* PY79.

## Materials and Methods

### Bacterial Strains, Conditions, and Culture

*E. coli* DH5α was used as a host for cloning throughout the study. The plasmid DNA used to transform *B. subtilis* was propagated in the *recA*-proficient strain *E. coli* BL21(DE3) pLysS. *B. subtilis* PY79 (wild type, prototrophic derivative of *B. subtilis* 168) [48] was used as the bacilysin producer strain. *S. aureus* ATCC 9144 was used as the bioassay organism for bacilysin detection. *B. subtilis*, *E. coli*, and *S. aureus* strains were usually grown in Luria Bertani (LB) medium at 37ºC. The PA medium [30] was used for bacilysin production. Antibiotic concentrations employed in this study for direct selection were as follows: kanamycin (Km) (5 μg /ml), chloramphenicol (Cm)(5 μg /ml), and ampicillin (Amp) (100 μg/ml). 

### Construction of the Homology Template for Introduction of the Strong SD Sequence

In this study, as a CRISPR/Cas9 plasmid, pJOE9958.1 was used. It is a derivative of the temperature-sensitive shuttle plasmid pJOE8999 [29]. Like pJOE8999, it is a single-plasmid system based on the *cas9* wild-type gene under the control of the mannose-inducible promoter P_manP_. Apart from JOE8999, among the two *Sfi* cutting sites, pJOE9958.1 bears the *ccdB* positive selection marker, which acts by killing the background of cells with no insert DNA, only cells containing a recombinant plasmid giving rise to viable clones in the F- *E. coli* cells (Fig. S1).

A homology template (932 bp) was constructed by employing an overlap extension PCR strategy by using *B. subtilis* PY79 chromosomal DNA as a template and High Fidelity Q5 DNA polymerase (New England Biolabs, USA). For this, a 313-bp upstream region from the *bacA* start codon was amplified via the first PCR reaction, which was performed by using the oligonucleotides: 5’-AAGGCCAACGAGGCCattaggttctgctttaat-3’(P1) as the forward primer containing the restriction site for *SfiI* (underlined residues) and 5’-**GAGTTTGTCCTCCTTAAGC**ttttaaaattttaagtaaattttatccagtgg-3’ (P2) as the reverse primer containing the *bacA*_strongRBS_ (bold residues). Then, 615 bp of the *bacA* open reading frame was amplified via the second PCR reaction by using the oligonucleotides: 5’-CTTAAAATTTTAAAA **GCTTAAGGAGGACAAACTC**atgattatattggataatagc-3’(P3) as the forward primer containing residues complementary to the reverse primer used in the first PCR reaction (bold and underlined residues), and 5’-AAGGCCTTATTGGCCttagttttcatcatcaacgtc-3’ (P4) as the reverse primer containing the restriction site for SfiI (underlined residues). Subsequently, the first and the second PCR products were aligned and amplified via the third PCR reaction using the oligonucleotides: 5’-AAGGCCAACGAGGCC ATT-3’(P5) as the forward primer and 5’-AAGGCCTTATTGGCCTTAGTTTTCATC-3’ (P6) as the reverse primer. Subsequently, the prepared 932 bp homology template (HT) was cut with *Sfi1* and ligated with the *Sfi1* digested pJOE9958.1 vector to construct pJOE9958.1.*bacA*_strongRBS_ (Fig. S1).

sgRNA for targeting of *bacA* RBS site was designed with the CRISPR/Cas9 online prediction tool CCTop [31]. *Bacillus subtilis* subsp. *subtilis* *168* genome sequence (Accession CP010052) was selected as the reference. Target site and core lengths were adjusted to 20 bp and 12 bp, respectively. A total of five mismatches were allowed. This sequence was synthesized into two complementary oligonucleotides with custom 5′ overhangs (TACG and AAAC) to produce compatible ends to the *BsaI*-treated vector pJOE9958.1.*bacA*_strongRBS_ and inserted into the *BsaI*-cut pJOE9958.1.*bacA*_strongRBS_ to get pJOE9958.1.*bacA*_strongRBS.sgRNA_ (Fig. S1). Proper insertion of sgRNA and the homology template at the pJOE9958.1.*bacA*_strongRBS.sgRNA_ was further confirmed via sequencing.

### Insertion of the Strong SD Sequence into the Upstream of *bacA* Start Codon

To transform *B. subtilis* PY79, pJOE9958.1.*bacA*_strongRBS.sgRNA_ was first propagated in the *recA*-proficient strain *E. coli* BL21(DE3)pLysS to prepare the multimeric plasmids. The competent *B. subtilis* PY79 cells were then transformed with pJOE9958.1.*bacA*_strongRBS.sgRNA_, and transformants were selected on the LB agar plates containing Km and 0.2% mannose for induction of *cas9* under the control of P_manP_ at 30°C. Colonies appearing within two days were streaked on LB plates containing Km and incubated at 30°C overnight. To cure their plasmids, isolates were plated on LB agar without antibiotics and incubated at 50°C; the next day, they were streaked on LB plates to obtain single colonies at 42°C. Finally, the colonies were checked for plasmid loss by transferring single colonies to LB agar plates with Km and incubating them overnight at 42°C.

### Bacilysin Production

Fresh culture of *B. subtilis* strains grown on LB agar were used to inoculate 10 ml of PA medium and cultivated overnight by shaking (200 rpm) at 37°C. This overnight culture was used to inoculate 100 ml of fresh PA medium to an initial OD_600_ value of 0.1. The cultures were incubated at 37°C for 24 h by shaking at 200 rpm. The bacilysin activity in culture fluids was determined by the paper disc-agar diffusion assay using *Staphylococcus aureus* ATCC 9144 as the assay organism. Briefly, 20 μl of culture supernatants was embedded into 6.0-mm paper disks, which were transferred onto bioassay plates containing assay organisms and incubated for 16 h at 37°C. Bacilysin activity was estimated as previously described [32].

### Ultra-Performance Liquid Chromatography-Mass Spectrometry (UPLC-MS) Analysis

One-fourth volume of butanol was used to extract 200 μl of culture supernatants, which was subsequently lyophilized to dryness. Samples were analyzed by UPLC-MS (Waters Acquity UPLC H-Class and Waters Synapt G2-Si HDMS, USA) at the positive detection mode after dissolving in 10 L of a solvent mixture (H2O: 5% CH3CN+ 1% HCOOH). Then, 7.5 μl of the sample was loaded onto a Waters Acquity Peptide BEH C18 column (2.1 mm × 100 mm) equilibrated in a solution mixture (1% CH3CN + 0.1% HCOOH + H2O) with a flow rate of 0.2 ml/ min, and a column temperature that was kept at 65°C. Bound compounds were eluted using the following gradient: Solution A (0.1% HCOOH:H2O) and Solution B (CH3CN): 1% Solution B for 1 min, 1–40% of Solution B over 10 min, 40–80% Solution B over 1 min and kept for 2 min, 80–1% of Solution B over 50 s, and re-equilibration with 1% Solution B for 10 min before loading the following sample.

### RT-qPCR Analyses

For total RNA preparation, the fresh culture of *B. subtilis* PY79 and HWA strains grown on LB agar were used to inoculate 10 ml of PA medium and cultivated overnight by shaking at 200 rpm at 37°C. This overnight culture was used to inoculate 100 ml of fresh PA medium to an initial OD_600_ of 0.1and grown for 24 h by shaking at 200 rpm at 37°C. The samples were collected at the early and late stationary phases of growth (corresponding to 18 h and 24 h of growth, respectively) for total RNA extractions. Total RNA from the samples was extracted using the Qiagen RNeasy Mini Kit (Germany). RNA quality and quantity were determined using the Nanodrop Lite spectrophotometer (Thermo Fisher Scientific, USA). As recommended by the manufacturer, 2 μg of total RNAs was reversely transcribed by using a Transcriptor cDNA Synthesis Kit (Roche, Switzerland) with random hexamer primers (60 μM). Ampliﬁcation and detection of qPCR products were performed with the SYBR Green Master Mix Kit (Roche) and a Light Cycler 480 (Roche) instrument. Then, 2 μl of synthesized cDNAs was used as the template in a 20 μl real-time PCR mixture. The PCR amplification conditions were as follows: preheating at 95°C for 5 min followed by 45 cycles of 95°C for 5 s, 52°C for 20 s, and 72°C for 30 s. Melting curve analysis was used to monitor the speciﬁcity of the reaction. The relative transcription level of the target gene was quantified by the 2^−ΔΔCT^ method [42] using the *sigA* gene as the internal control. Two independent qPCR experiments were performed, with each experiment containing three biological and two technical replicates. For statistical analysis, Student’s *t*-test was performed and statistical significance was reported as ns (no significance, *p* > 0.05), *(*p* < 0.05) and **(*p* < 0.01).

## Results and Discussion

### Strong RBS Sequence Design

In this study, to analyze the impact of the RBS region on the enhancement of bacilysin production, the canonical strong SD sequence 5’-TAAGGAGG-3’ was combined with “ACAAACTC”-8nt spacer sequence, thus resulting in the 5’-GCT**TAAGGAGG**ACAAACTC**ATG-3**’ RBS sequence, named *bacA*_strongRBS_. “ACAAAC” was selected as an optimized spacer sequence typically used within the *B. subtilis* IPTG inducible promoter P_hyperspank_. Moreover, it was also used as the standard spacer sequence in work performed by Guiziou *et al*. [33]. However, we preferred to keep the spacer length at 8 nt as that was found to be optimum in previous studies [14,20,21]. Therefore “ACAAAC” sequence was furthered with the original “TC” nucleotides upstream of the ATG start codon. Also employed was the RBS Calculator (https://www.denovodna.com/software) [18], which is widely used to evaluate the translation initiation rate of RBS sequences. Before in vivo testing, the expected translation initiation rate of *bacA*_strongRBS_ and *bacA*_nativeRBS_ (5’-GATTGGTTGGTGCTC**ATG**-3’) was predicted by using the RBS Calculator, and the results are given in Fig. S2. The predicted translation initiation rate of *bacA*_strongRBS_ was found to be 219113,65 au, which was approximately 93-fold higher than that of the native RBS sequence from *bacA* (2347,58 au), verifying our hypothesis about the weak performance of the native RBS region of the *bac* operon along with the positive impact of a strong RBS. 

### *bacA*-5’UTR Editing

CRISPR/Cas9 single-plasmid systems allowing the introduction of the gRNA and the repair template on a single-plasmid system were also successfully adopted for *B. subtilis* genome editing and successfully applied for gene-operon deletion and mutation repair [29, 34, 35]. Thus, for *bacA*-RBS editing, we preferred to use a CRISPR/Cas9 single-plasmid system based on the *cas9* wild-type gene under the control of the mannose-inducible promoter P_manP_, pJOE9958.1 [29].

In this study, a 932 bp homology template, including 19 nt *bacA*_strongRBS_ flanked by the -298 bp upstream and 615 bp downstream of the *bacA* start codon, was amplified by overlapped-extension PCR (Fig. 1B). The upstream sequence laying at -28 and -8 bp position from the *bacA* start codon (Fig. 1B) was selected as a 20 nt spacer sequence for guiding the Cas9 to the upstream of the *bacA* region by using the software CCTop [31] due to its lack of an off-target score. The generated pJOE9958.1.*bacA*_strongRBS.sgRNA_ was used to transform *B. subtilis* PY79 competent cells, and transformants were selected on the LB agar plates containing Km and 0.2% mannose for induction of *cas9* under the control of P_manP_ at 30°C. Twenty-one colonies appeared within 2 days and were restreaked on LB plates containing only Km and incubated at 30°C. Before plasmid curing, Km^R^ colonies were screened for bacilysin phenotype to eliminate the potential bacilysin nonproducers (Bac^-^), since Bac^-^ candidates are likely to have arisen from cells repaired through the non-homologous end-joining (NHEJ) pathway. NHEJ and the homology-directed repair (HDR) pathways are the two critical processes for repairing double-strand breaks (DSBs) in genomic DNA. For this, high-throughput bacilysin screening was employed by placing the resulting colonies with toothpicks on bioassay plates containing *S. aureus* ATCC9144, which were then incubated overnight at 30°C. As shown in Fig. S3, of the 21 Km^R^ colonies, only 3 colonies exhibited significant antibacterial activity against *S. aureus*, suggesting that the efficiency of HDR remained too low for repairing Cas9-mediated-DSBs, and that most of the colonies in our study were most likely derived from the NHEJ pathway. This finding contradicted prior studies that found the NHEJ pathway in Bacillus species inefficient and inactive in growing cells, and generally more active in the stationary phase or during sporulation [36,42,43]. Therefore, we tested whether the error-prone NHEJ pathway plays any role in DSB in *B. subtilis* PY79, pJOE9958.*bacA*_sgRNA_, which only contains a 20 nt spacer sequence directed against *bacA* and no homology template, allowing surviving colonies to be generated only through the NHEJ pathway. This plasmid was quickly created by deleting the homology template from the control plasmid, pJOE9958.1.*bacA*_strongRBS.sgRNA_, and used in parallel with the control plasmid for *B. subtilis* PY79 transformation. Compared to the control plasmid, which produced only 46 ± 18 transformants with a transformation efficiency of 5.5 ± 2.1 transformants/μg DNA, pJOE9958.*bacA*_sgRNA_ produced 147 ± 13 transformants with a transformation efficiency of 19.6 ± 1.7 transformants/μg DNA. These results confirm that Cas9-caused DSB was primarily repaired by the error-prone NHEJ rather than HDR in our work. 

As a further step, to generate the plasmid-cured cells, only Km^R^ and Bac^+^ colonies were plated on LB plates without Km to single colonies at 50°C overnight as a nonpermissive temperature for plasmid replication and then restreaked to single colonies on LB plates at 42°C. The colonies were then screened for the loss of Km^R^ phenotype, and roughly 42% of all colonies examined had lost the plasmids. This efficiency was lower than expected for pJOE8999 derivatives in *B. subtilis* strains since highly efficient plasmid curing rates of 83-90% have been reported previously for those derivatives [29]. However, the curing rate of the pJOE9958.1 derivative used in this study was still adequate for the generation of plasmid-cured cells with the desired modification as seen below.

**Fig. 2:**
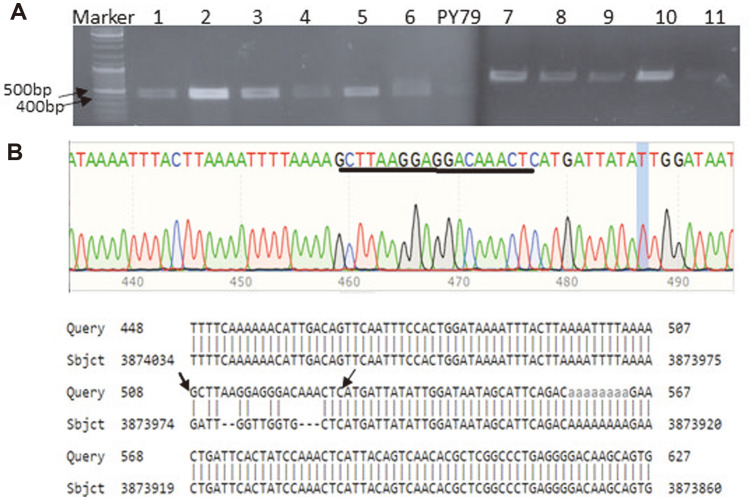
(A) Colony PCR analysis performed with Km^S^ and Bac^+^ colonies. 5’-AGCTTAAGGAGGAACAACTC- 3’(*bacA*_strongRBS_ sequence) was used as the forward primer and 5’-GGTAAAATATTTTATTAAA-3 (reverse complement to the 402-420 nt region of *bacA*) was used as the reverse primer. M: Marker (*EcoRI* and *Hind*III digested Lamda DNA). (**B**) DNA sequence analysis of a Km^S^ and Bac^+^ mutant. The introduced *bacA*_strongRBS_ sequence “GCTTAAGGAGGACAAA” was underlined in its DNA sequencing chromatogram and indicated with arrows in its BLAST analysis result (https://blast.ncbi.nlm.nih.gov/Blast.cgi). In the Blast analysis, *B. subtilis* PY79 (taxid:1415167) was used as the reference geneome.

In the last stage, to find mutant candidates possessing the substitution of the *bacA*_strongRBS_ sequence with the *bacA*_nativeRBS_ sequence, 11 randomly selected Km^S^ (plasmid-cured) transformants were tested by colony PCR by using the oligonucleotide 5’-AGCTTAAGGAGGAACAACTC-3’ (specific to substituted strong RBS sequence) as the forward primer and the oligonucleotide 5’-GGTAAAATATTTTATTAAA-3 (reverse complement to the 402-420 nt region of *bacA*) as the reverse primer. Of those, 10 exhibited the expected PCR fragment of about 440 bp (Fig. 2A). DNA sequencing results of the one candidate, selected randomly among the PCR^+^ colonies, indicated that the *bacA*_strongRBS_ sequence was successfully replaced with the *bacA*nativeRBS sequence as expected (Fig. 2B).

### Screening of Bacilysin Activities of *bacA* strong_RBS_ Candidates

**Fig. 3:**
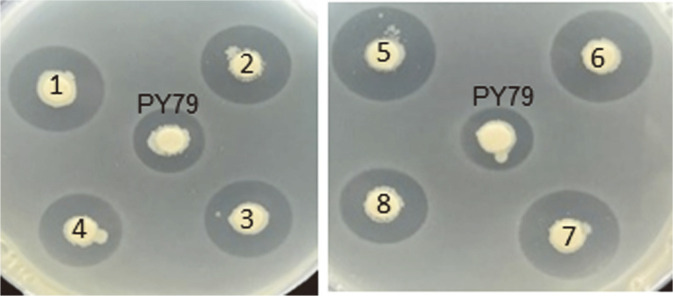
Bacilysin titers in mutants harboring strong-RBS and the parental strain (PY79). Strains were cultivated in PA medium for 16-18 h at 37°C via shaking at 200 rpm, and bacilysin levels in their culture fluids were detected by paper diskdiffusion assay using *Staphylococcus aureus* ATCC 9144 as the assay organism.

To analyze the effect of the strong RBS insertion, the eight candidates comprising the strong RBS mutant verified by sequence analysis described above, together with the other seven candidates (randomly selected among the PCR^+^ colonies), were cultured in PA media for 16-18 h, and the bacilysin level in their culture fluids was detected by paper disk-diffusion assay. Compared to PY79, each of the examined mutants produced more bacilysin in which there were no considerable changes in their increased bacilysin titers (Fig. 3), thus demonstrating that the strong RBS substitution resulted in increased bacilysin production. The antibacterial activity exhibited by mutants was entirely due to bacilysin. Because their antibacterial activities were completely suppressed by supplementing the bioassay plate with N-acetylglucosamine known as a particular antagonist of bacilysin activity [37] (Fig. S4). Finally, one of the *bacA*strongRBS mutants (numbered 5 in Fig. 3) was chosen to represent strong RBS-carrying mutants and named *B. subtilis* HWA for further research.

### Comparison of the Intracellular mRNA Level of the *bac* Operon in HWA with PY79

In bacteria, transcription and translation are coupled, and changing the nucleotides at 5’UTR can influence both transcriptional and translational processes [40, 49]. According to research by Berg et al. [49], point mutations within the 5′UTR not only impact translation rates but also influence transcript abundance. Consistently, a study by Eriksen *et al*. [40] indicates that RBS strength can impact the degree of protein synthesis also by presumably influencing the stability of the mRNA due to increased ribosome occupancy, occluding binding and cleavage sites on the transcript. Thereby, mRNA transcripts may be more protected from degradation by exo- and endonucleases, increasing the absolute level of mRNA.

**Fig. 4:**
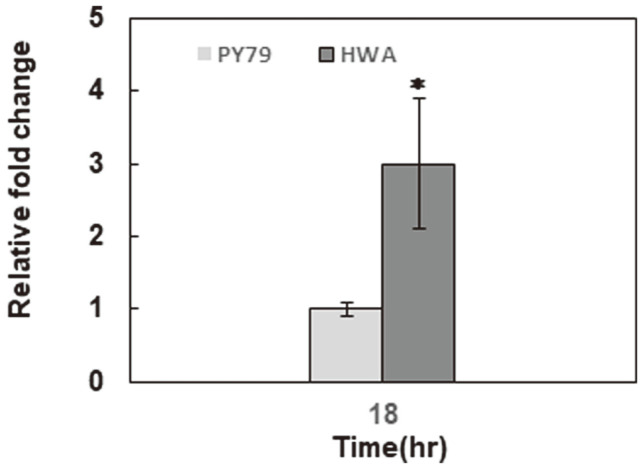
Transcriptional analysis of the *bac* operon in HWA and PY79. The relative transcript level of *bacB* as the second gene in the *bac* operon was detected in HWA and PY79 cells via RT-qPCR at the early stationary phase of growth (corresponding to 18 h). Error bars indicate the standard errors of the means of the two independent qPCR experiments performed with three biological and two technical replicates. Asterisk (*) indicates statistically significant differences from the parental strain PY79 with (*p* < 0.05) according to paired Student’s t-test.

**Fig. 5:**
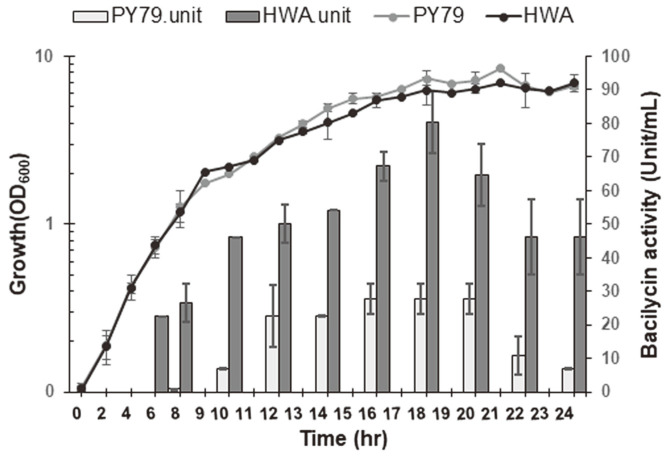
Growth and bacilysin activity profiles of HWA and PY79. Strains were grown in PA medium for 24 h at 37°C via shaking at 200 ×*g* and bacilysin activity in their culture fluids was detected by paper disk-diffusion assay. Error bars represent the standard deviation of the means of three independent experiments

In light of these facts, to highlight the effect of 5’UTR modification via the strong RBS substitution at the intracellular mRNA level, the relative transcript amounts of the *bac* operon were detected in HWA and PY79 via RT-qPCR at the early and late stationary phases of growth (corresponding to 18h and 24h of growth, respectively). As shown in Fig. 4, we first compared the relative transcript amounts of the *bac* operon in HWA and PY79 samples at 18 h of growth with the maximum bacilysin activity detected at 18 h of incubation in both strains (Fig. 5). HWA harboring strong RBS showed a significant change in the *bac* operon mRNA levels, as evidenced by a 3-fold higher level in the early stationary phase. Furthermore, its transcript level in HWA was detected as 25 quantitative cycles (Cq) in the early stationary phase (18 h) and 2l Cq in the late stationary phase (24 h), in contrast with PY79 (29 Cq at 24 h) (Fig. S4), revealing that a high transcript level of the *bac* operon in HWA seems to be maintained throughout the stationary phase. Conclusively, all these results indicate that strong RBS substitution also significantly improved the mRNA stability of the *bac* operon. 

### Comparison of Bacilysin Production Performance of HWA with PY79

**Fig. 6:**
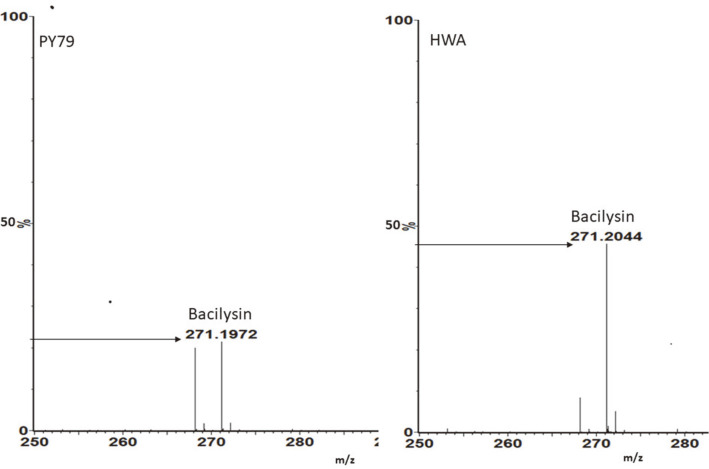
UPLC-MS spectrum of HWA and PY79 culture extracts. UPLC-MS analyses were performed in positive detection mode. Bacilysin [M + H]^+^ at m/z 271 was detected in their culture extracts.

To further verify the interaction between the strong RBS substitution and over-production of bacilysin, HWA and the parental strain PY79 were cultivated in PA medium at 37°C with shaking for 24 h; their growth and bacilysin production levels were measured at intervals throughout their cultivation periods. As shown in Fig. 5, there was no considerable change in the growth and the cell density dependent-bacilysin accumulation profiles of HWA in comparison to PY79 [38], in which the maximum bacilysin activity was still detected at the early stage of the stationary phase (corresponding to 16-18 h of cultivation). The most striking finding was that HWA exhibited over-production of bacilysin at all growth stages by peaking at 18 h of incubation. The bacilysin titer of HWA was 2.87-fold higher than the wild-type strain, with bacilysin activity rising from 27.9 ± 4.5 U/ml (in parental strain) to 80.3 ± 9.1 U/ml (in HWA) at the end of 18 h of cultivation. UPLC-MS analysis further confirmed the significant change in the bacilysin activity of HWA in the 16-18 h culture. The result was higher relative abundance levels of the peak m/z of 271 (M + H)^+^ corresponding to the mass of bacilysin relative to that of PY79 (Fig. 6).

All these findings are highly significant as the results of the first study on 5’UTR editing employed to improve bacilysin production in its producer strain have revealed that extensive RBS engineering seems to be a promising strategy for enhancing bacilysin production. In the present study, we could only test a single strong RBS sequence. In a further study, the nucleotide (nt) compositions around the nucleotide sequences up and downstream from the SD site should optimize since those regions can also affect the translation efficiency by forming the mRNA secondary structures, which can occlude the RBS and thereby restrict translation initiation [18, 39]. Furthermore, in this study, a spacer length of 8 nt was used as the optimum spacer length based on previous reports [14, 21] in which variants with spacer lengths of 7-9 nt were among the best-producers, as was the case in their in silico predictions. But, besides its nt composition, the optimum length of a spacer can also change according to the gene of interest, and in turn, a particular RBS can be strong when used with one gene but weak when used with another gene [18, 21, 33, 45]. In addition, it is difficult to predict the optimal strength of RBSs for genes of interest in a biosynthetic pathway or an operon, and RBS engineering by creating libraries with different strengths and sequences is usually employed as a popular and efficient approach to precisely tune gene expression at the translational level [44-46]. For instance, Jin *et al*. [46] successfully engineered *B. subtilis* 168 to produce specific low-molecular-weight hyaluronan (LMW-HA) directly from sucrose. In that study, RBS optimization by using a series of RBS variants with different translation efficiencies was employed to fine-tune the level of leech hyaluronidase (LHAase), allowing the production of LMW-HAs with a molecular weight range of 2.20 × 10^3^ – 1.42 × 10^6^ Da. A genetic toolbox comprising libraries of promoters, ribosome binding sites (RBS), and protein degradation tags to precisely tune gene expression in *B. subtilis* was engineered by Guiziou *et al*. [33], which further supported the efficiency of promoter and RBS libraries for fine-tuning gene expression in *B. subtilis*. In a very recent study [47], the recombinant production of TypeI L-asparaginase from *Bacillus licheniformis* Z-1 (BlAase) in *B. subtilis* RIK 1285 was significantly improved by the combination of library-based promoter and RBS engineering strategies. Conclusively, a library-based RBS optimization could be an effective approach for further enhancing and fine-tuning the bacilysin biosynthetic pathway in *B. subtilis*.

In this study, by using a CRISPR/Cas9 single-plasmid system, the putative RBS region of the *bac* operon (*bacABCDEF*) was edited by introducing the canonical SD sequence (TAAGGAGG) at 8 nt optimum spacing from the *bacA* start codon (AUG). Since the inspection of the upstream region of the *bac* operon did not reveal a core SD sequence with an ideal spacing (7-9 nt), it is suggested that *bacA*-RBS editing most likely has a positive impact on the production of bacilysin in its native producer. As expected, 5’UTR modification via the strong RBS substitution was associated with a remarkable increase in bacilysin production. Furthermore, the transcript level of the *bac* operon was significantly increased, particularly in the late stationary phase, implying that a strong RBS substitution to the *bac* operon could improve not only its translation initiation efficiency but also its mRNA stability. Overall, these data revealed that extensive RBS engineering could be a promising key option for improving bacilysin production in native producers. Our study represents the first instance of RBS editing employed to improve bacilysin production in its native producer, *B. subtilis* PY79. Moreover, our results will help to further enhance bacilysin production in native strains.

## Supplemental Materials

Supplementary data for this paper are available on-line only at http://jmb.or.kr.
