# Supplementary material for: Improvement of Bacilysin Production in Bacillus subtilis by CRISPR/Cas9-Mediated Editing of the 5’-Untranslated Region of the bac Operon
Source: J Microbiol Biotechnol. 2022 Dec 13;33(3):410–8. doi: 10.4014/jmb.2209.09035 (PMC10084748; doi:10.4014/jmb.2209.09035)
Supplement: Supplementary file 1 [file jmb-33-3-410-supple.pdf]

J. Microbiol. Biotechnol.
https://doi.org/10.4014/jmb.2209.09035

SUPLEMENTARY MATERIALS

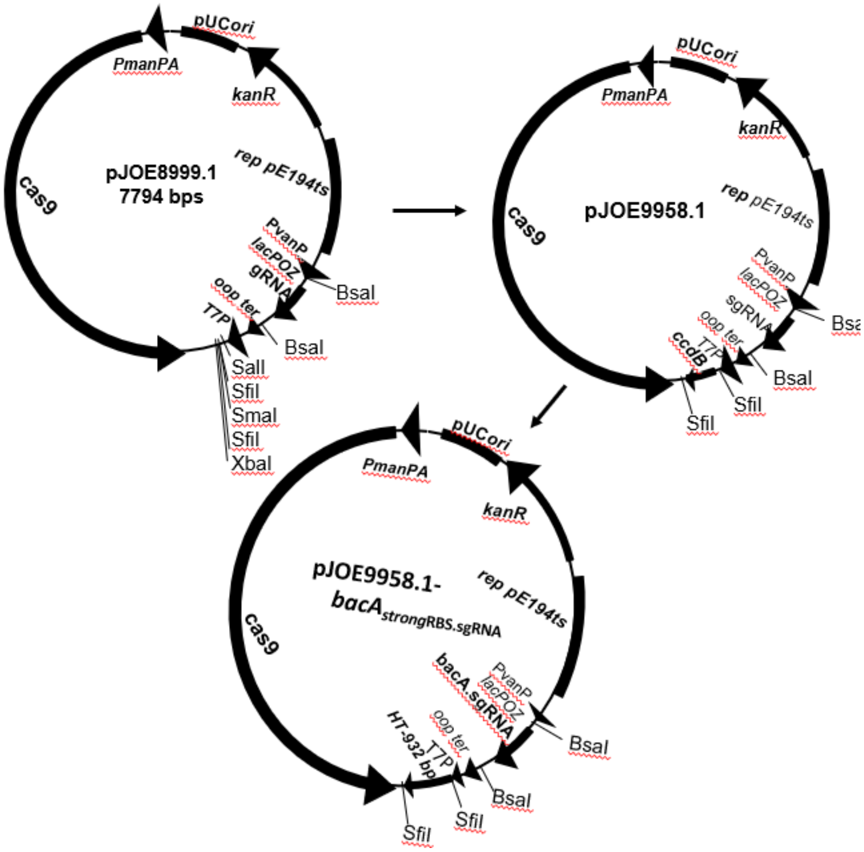

Fig.S1. Maps of pJOE9958.1, pJOE99.58.1 and pJOE9958.1-*bacA*$_{\text{strongRBS.sgRNA}}$ constructed in this study.

(A)

Results

Predicted translation initiation rates at start codons (click start codons for more details)

AUAAAAUUUACUUAAAAUUUUAAAAGCUUAAGGAGGACAAACUCAUGAUUAUAUUGGAUAAUAGCAUUCAGACAAAAAAAAGAACUGAUUCACUAUCCAAACUCAUUACAGUCA

Translated Open Reading Frames

Free Energy Calculations and Accuracy Warnings

Pre-cutoff value: 100 nt
Maximum bulge in aSD/SD: nt
RNA Energy Model: rna_andronescu2007.par
Post-cutoff value: 35 nt
Suboptimal energy gap in aSD/SD: 8 kcal/mol
System Temperature: 37°C
RNA dangles value: 0
Ribosome footprint: 13 nt
3' end of 16S rRNA: 3'-ACCUCCUUU-5'

Start Position ↑	Frame	Translation Rate	ΔG_{total}	$\Delta G_{mRNA\text{-}rRNA}$	$\Delta G_{spacing}$	$\Delta G_{stacking}$	$\Delta G_{standby}$	ΔG_{start}	ΔG_{mRNA}
44	+2	219113.65	-11.51	-11.28	0.20	0.00	0.00	-2.76	-2.41
53	+2	85.23	5.94	-12.75	14.28	0.00	0.00	1.81	-3.70

(B)

Results

Predicted translation initiation rates at start codons (click start codons for more details)

AUAAAAUUUACUUAAAAUUUUAAAAGAUUGGUUGGUGCUCAUGAUUAUAUUGGAUAAUAGCAUUCAGACAAAAAAAAGAACUGAUUCACUAUCCAAACUCAUUACAGUCA

Translated Open Reading Frames

Free Energy Calculations and Accuracy Warnings

Pre-cutoff value: 100 nt
Maximum bulge in aSD/SD: nt
RNA Energy Model: rna_andronescu2007.par
Post-cutoff value: 35 nt
Suboptimal energy gap in aSD/SD: 8 kcal/mol
System Temperature: 37°C
RNA dangles value: 0
Ribosome footprint: 13 nt
3' end of 16S rRNA: 3'-ACCUCCUUU-5'

Start Position ↑	Frame	Translation Rate	ΔG_{total}	$\Delta G_{mRNA\text{-}rRNA}$	$\Delta G_{spacing}$	$\Delta G_{stacking}$	$\Delta G_{standby}$	ΔG_{start}	ΔG_{mRNA}
27	+0	80.55	6.06	1.16	0.00	0.00	0.00	1.81	-3.32
31	+1	65.83	6.51	-0.19	0.68	0.00	0.00	1.81	-4.91
34	+1	305.82	3.10	-0.92	0.00	0.00	0.00	-0.42	-5.13
40	+1	2347.58	-1.43	-5.93	0.68	0.00	1.06	-2.76	-6.00
49	+1	98.67	5.61	-1.93	0.68	0.00	0.00	1.81	-6.00

Fig. S2. Expected translation initiation rates of *bacA*$_{strong\text{RBS}}$ (A) and *bacA*$_{native\text{RBS}}$ (B) predicted by using RBS calculator (https://www.denovodna.com/software/)

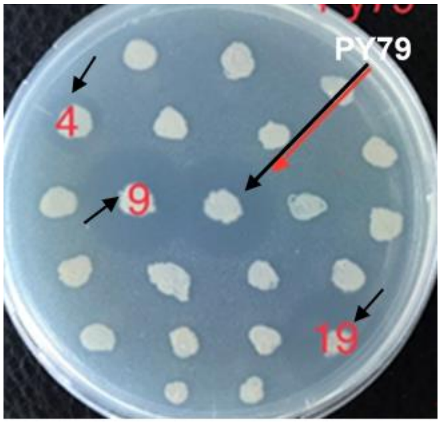

Fig. S3. High throughput screening of KmR transformants containing pJOE9958.1.bacA$_{strongRBS.sgRNA}$ on bioassay plates containing *S. aureus* ATCC 9144 for bacilysin activity. Colonies were directly transferred on bioassay plates with toothpicks and incubated for 16 hrs at 37°C. The wild-type bacilysin producer *B. subtilis* PY79 was used as the positive control.

+10mM N-acteylglucosamine

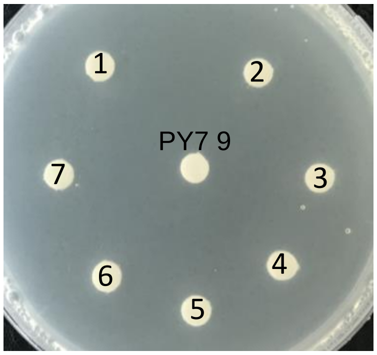

Fig. S4. Culture fluids of mutants harboring strong-RBS and the parental strain (PY79) screened on the bioassay medium containing *S. aureus* ATCC 9144, and 10 mM N-acetylglucosamine.

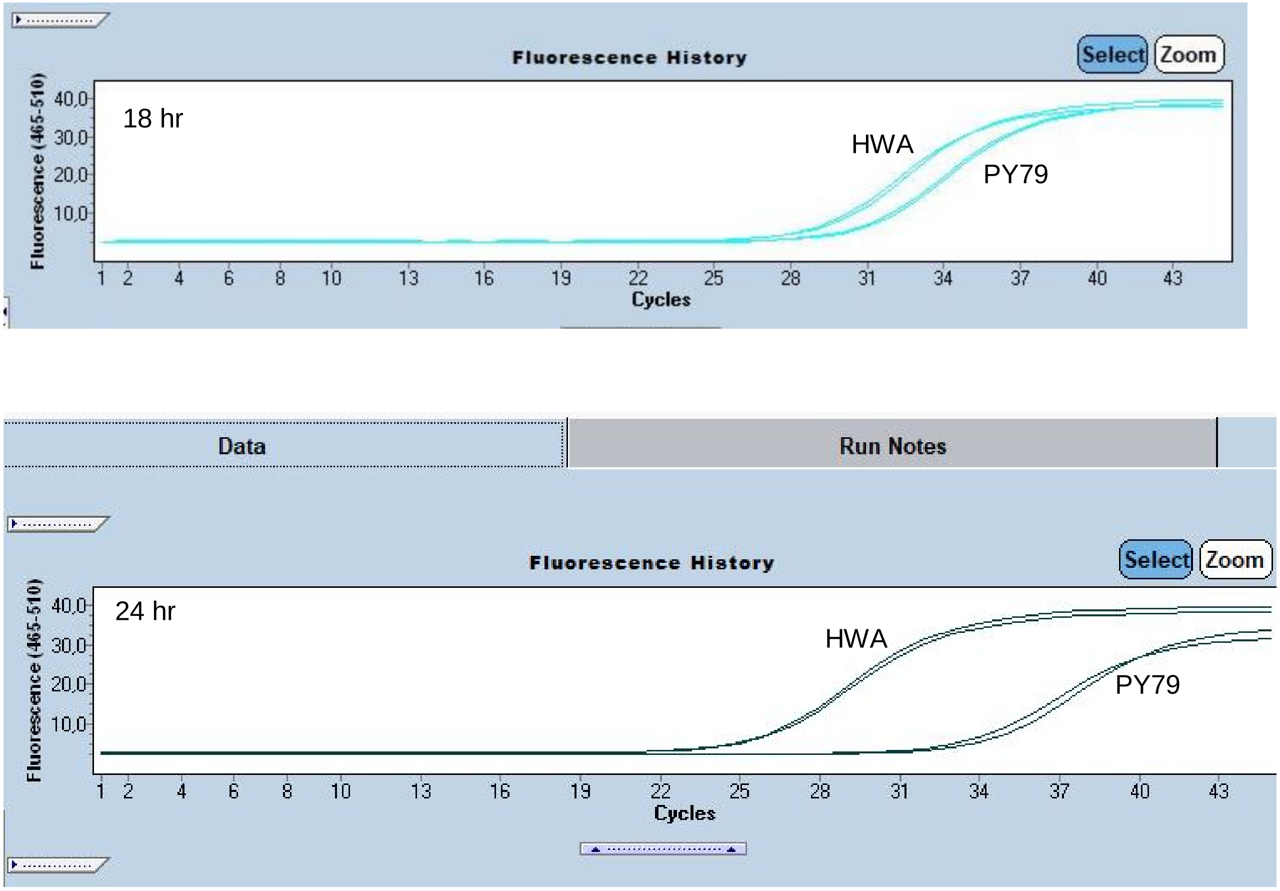

Fig. S5. RT-qPCR quantitative Cycle (Cq) values of *bacB* detected in HWA and PY79 cells at the end of 18 hrs and 24 hrs of growth.
